# Making the most of rodent tumour systems in cancer drug discovery

**DOI:** 10.1038/sj.bjc.6690261

**Published:** 1999-04

**Authors:** M C Bibby

**Affiliations:** Clinical Oncology Unit, University of Bradford, Richmond Road, West Yorkshire, BD7 1DP, UK


*British Journal of Cancer* (1999) **79**(11/12), 1633–1640
© 1999 Cancer Research Campaign
Article no. bjoc.1998.0261

Review

### Making the most of rodent tumour systems in cancer drug discovery

**MC Bibby**

Clinical Oncology Unit, University of Bradford, Richmond Road, Bradford, West Yorkshire, BD7 1DP, UK

It is clear that even after almost half a century of intensive effort to develop effective treatments for common solid cancers there is still some way to go before a major impact on survival of patients with these malignancies is achieved. Much of the paucity of success is blamed on the lack of appropriate models and there is a commonly held belief amongst cancer researchers that transplantable tumours in rodents are sensitive to drug therapy, are easy to cure and therefore not predictive of responses in humans. It is true that, in the past, when one considers the large number of compounds evaluated, murine tumour models have identified only a limited number of clinically useful agents and not a single cancer-specific drug has resulted from a murine tumour screen. It is easy with hindsight to blame this disappointing lack of really effective therapies on inappropriate test systems and screening strategies but, although drug discovery from rodent systems has been sparse, in reality, an enormous contribution to cancer therapy principles has emanated from such studies. This work really began in the mid 1950s when the National Cancer Institute (NCI) in the USA initiated a large-scale anti-cancer drug-screening programme using three murine tumour models: sarcoma 180, L1210 leukaemia and carcinoma 755 (Plowman et al, 1997). Of major significance in the early days was the work of Skipper and colleagues who, by the use of experimental leukaemia models, established the principle of survival/inoculum relationship (Skipper et al, 1957) and also the concept of fractional cell kill, i.e. that the percentage of the tumour cell population killed by a given dose of drug was relatively constant (Skipper et al, 1964). They also described a close relationship between dose level and percentage of leukaemic cell population killed, i.e. a dose–response relationship. Other useful therapeutic strategies were first established in rodent systems. The principle of optimum scheduling was first described by Goldin et al (1956) using the L1210 leukaemia. Combination chemotherapy and the concept of therapeutic synergism was first investigated in rodent models (Goldin and Mantel, 1957) as was combining the modalities of surgery and chemotherapy – adjuvant chemotherapy (Martin, 1981).

Many of the early experimental studies utilized murine leukaemias which grew very rapidly, had a high growth fraction and proved to be sensitive to a number of agents that were subsequently shown to have more activity against leukaemias and lymphomas than against solid carcinomas or sarcomas and to be toxic to the bone marrow (Muggia, 1987). A number of investigators believed that the use of more appropriate, slower growing tumour models of solid cancers, and adoption of clinically relevant end-points, would improve the usefulness of preclinical studies, particularly for agents with potential activity against the common solid cancers. Corbett et al (1987) pointed out that most of the agents that had entered the clinic at that time had poor, or no, activity against the majority of transplantable solid tumours in mice but they also suggested that, as negative findings are rarely reported, the casual reader of the drug discovery literature may have gained the inaccurate impression that transplantable tumours in mice are highly vulnerable to a large proportion of agents that make the clinic.

*Received 10 July 1998*
*Revised 24 August 1998*
*Accepted 16 September 1998*

*Correspondence to:* MC Bibby

### THERAPEUTIC INDEX

The importance of addressing the therapeutic index of investigational agents has been stressed by many groups over the years, including our own (Double and Bibby, 1989) and we have advocated a more thorough preclinical evaluation of new cytotoxic entities which assesses efficacy and normal tissue toxicity in the same setting (Bibby et al, 1988). Taking mitozolomide (Stevens et al, 1984) as an example compound that had recently entered clinical trial, we evaluated anti-tumour activity in a limited panel of refractory murine tumours but we also investigated normal tissue toxicity at the active doses. Mitozolomide showed a poor therapeutic ratio in this test system with modest anti-tumour effects being seen close to maximum tolerated dose (MTD) only. At this dose level there was very severe normal tissue toxicity in mice (Table 1). Mitozolomide is an example of (at that time) a novel chemical that showed exciting pre-clinical activity in some experimental tumour systems with cures in L1210 and P388 at half the LD10 but went on to behave poorly in the clinic due to unmanageable haematological toxicity. Although rodent studies do not

**Table 1** Bone marrow toxicity of mitozolomide assessed by spleen colony forming unit (CFU-S) assay in mice

| Donor | | Recipient | |
|---|---|---|---|
| Dose (mg $kg^{-1}$) | Radiation (Gy) | No. of bone marrow cells injected | CFU-S |
| – | 11.7 | $1.2 \times 10^4$ | 40 |
| 20 | 11.7 | $1.0 \times 10^5$ | 4 |
| 30 | 11.7 | $9.0 \times 10^4$ | 3 |
| 40 | 11.7 | $1.0 \times 10^5$ | 0 |

*n* = 6 mice per group. Data taken from Bibby et al 1988.

necessarily predict human organ toxicity, they do appear to be helpful in predicting effects in some organs, e.g. bone marrow. Hindsight has shown that mitozolomide was selected for clinical trial on the basis of results from inappropriate pre-clinical models that did not reflect the resistance of clinical disease, but the group has gone on to develop a useful less toxic analogue, Temozolomide (Newlands et al, 1997).

### THERAPEUTIC END-POINTS

In the early 1980s, Martin and colleagues discussed the significance of the methodology used in recording response (Martin et al, 1984). They assessed the importance of selecting an appropriate end-point in preclinical studies by comparing response rates in breast cancer patients to six agents and then evaluating the same six agents in a murine model of spontaneous breast cancer, the CD8F1 (Stolfi et al, 1988). The breast cancer patients responded only to melphalan, cyclophosphamide and 5-fluorouracil (5-FU). When the authors evaluated response in the murine system by percentage tumour inhibition, all six agents could be classified as active, whereas when they employed tumour regression as a measure of activity, as in the patients, only the three clinically active agents were effective (Table 2). Despite these observations cytotoxic agents have still progressed to clinical study on the basis of tumour growth delay in rodents at MTD and have often been shown to be ineffective in humans at doses that can be tolerated.

Obviously there are certain ethical considerations that need to be addressed when selecting model systems and end-points for in vivo studies. As long as appropriate guidelines for animal welfare are followed (e.g. UKCCCR; Workman et al, 1998), subcutaneous models of neoplasia are relatively straightforward to use and potential animal suffering can be monitored and appropriate humane end-points, such as tumour size, utilized. However, the use of ascites tumour models and survival end-points to investigate the activity of intraperitoneally administered agents is difficult to justify on scientific or ethical grounds. These should not be used for preclinical screening as they can result in undue suffering and provide little more information than well-designed in vitro assays. Lethality end-points should be avoided in general, even for models of systemic disease, and histological and/or metastatic colony counting techniques should be employed to assess anti-tumour effects. As modern therapeutic strategies develop, more effort is required to ensure suitable pharmacodynamic end-points are in place to assess whether or not a designed therapy is achieving its goal in vivo.

### IN VITRO CELL LINE PANELS

As a result of expanding knowledge, but also limited success, in the identification and development of drugs with useful activity in common solid cancers, drug-screening programmes in the NCI and elsewhere have evolved considerably over the years (Plowman et al, 1997) until in 1985 the NCI began to assess the feasibility of using human tumour cell lines for large-scale compound screening (Boyd, 1989). It is beyond the scope of this article to extol the virtues or otherwise of this approach to drug discovery but it is clear that, as a result of its inception, many interesting molecules are continually emerging and these will require appropriate in vivo evaluation. The NCI cell line panel has expanded since it was initially set up and there is ongoing development and characterization of potential new molecular targets. Although the initial strategy for the cell line screen was to develop a disease-orientated approach, the addition of the molecular characterization of the cell line panel opens up the possibility for target-orientated screening. The option to use genetically modified cell lines to address specific molecular targets at the in vitro stage is now available. This scientifically sound strategy of target-orientated drug development must be extended into in vivo evaluation protocols, so not only should in vivo studies be carried out in tumours derived from cell lines that were identified as sensitive in vitro, but it is imperative that adequate characterization of these models demonstrates that the molecular characteristics of the cell line are retained in the in vivo setting.

### PHARMACOKINETIC CONSIDERATIONS

Going from cell-free screening strategies and cell lines to in vivo studies represents a major step in the drug-discovery pathway and one very important requirement is knowledge of the pharmacokinetics of investigational agents at an early stage. Ideally, such studies should be carried out alongside efficacy investigations with a view to either selecting those agents with the best pharmaceutical properties or, alternatively, formulating or even modifying the active component to optimize the therapeutic effect.

**Table 2** Importance of clinically relevant endpoints in predictability of preclinical anti-tumour studies

| Drug | Human breast cancer | CD8F1 spontaneous breast tumours | | | |
|---|---|---|---|---|---|
| | | Inhibited (%)[a] | Assessment | Regression | Assessment[b] |
| Melphalan | + | 88 | + | 7/24 | + |
| Cyclo | + | 80 | + | 5/25 | + |
| FU | + | 79 | + | 13/60 | + |
| *N*-phosphon-acetylaspartate | – | 63 | + | 2/47 | – |
| Ara-c | – | 33 | + | 1/37 | – |
| 6-thioguanine | – | 52 | + | 0/45 | – |

[a]Statistically significant; [b]at least 50% reduction. Data taken from Stolfi et al 1988.

*© Cancer Research Campaign 1999*

### HOLLOW-FIBRE ASSAYS

One relatively new approach to in vivo drug testing has been developed at NCI utilizing human cell-lines growing in hollow fibres. It is intended as a method for prioritizing compounds for testing in xenografts. At present, 10 000 compounds are screened in vitro against the cell line panel each year by NCI and 8–10% are referred for in vivo testing (Plowman et al, 1997). For the hollow-fibre assay, tumour cells are inoculated into 1-mm internal diameter hollow-fibres that are heat-sealed and cut into 2 cm lengths. The fibres are maintained in in vitro culture for 24–48 h and then implanted intraperitoneally and subcutaneously into nude mice. Three different cell lines can be grown in two different sites in the same mouse. The effects of treatment are determined by MTT assay on removal of the fibres 6–8 days post-implantation. On the face of it, this technique would seem an efficient way of identifying lead compounds of promise because it requires relatively small expenditure and a limited quantity of test compound. Of course, as mentioned earlier, it cannot be assumed that expression of a particular target will be identical in vivo to that expressed when the same cells are grown in vitro or vice versa.

### TUMOUR CHARACTERIZATION

For several years our laboratory has been involved in studies of potential bioreductive drugs but in particular we have developed an interest in the role of the dimeric flavoprotein DT-diaphorase (DTD, NAD(P)H:Quinone acceptor reductase, E.C.1.6.99.2) in the activation of a number of quinone based anti-cancer drugs (Phillips, 1996). There is a wealth of literature indicating a good correlation between enzyme activity in cell lines and aerobic chemosensitivity to compounds like EO9, 3-hydroxy-5-aziridinyl-1-methyl-2(1H-indole-4,7-dione)-prop-β-en-α-o1 (Robertson et al, 1992; Smitskamp-Wilms et al, 1994; Collard et al, 1995; Fitzsimmons et al, 1996). However, when attempts were made to translate these in vitro observations into in vivo studies it became apparent that DTD activity in human tumour xenografts derived from cell lines did not usually mirror levels seen in vitro (Collard et al, 1995), indicating the necessity to further characterize xenografts for the appropriate target for which therapeutic molecules are being sought. In the case of DTD, antibodies are available (Segura-Aguilar et al, 1994) so it is possible to identify the precise location of the protein within tissue sections (Segura-Aguilar et al, 1994; Phillips et al, 1998), and similar immunolocalization can be carried out now with a whole host of target enzymes and other molecular targets. It is certain that the majority of targets currently being investigated for drug development strategies will be influenced by their local environment, so not only will there be differential target expression in different tumours but there will also be different expression in vivo from that seen in in vitro culture of the same tumour cells. Furthermore, it is clear that tumour site within the body can influence the expression of specific targets, e.g. P-glycoprotein. Fidler and colleagues investigated response to doxorubicin (DOX) or 5-FU in three tumour types growing in different anatomical sites (Dong et al, 1994; Fidler et al, 1994). Sensitivity to 5-FU did not alter with anatomical site but lung and tumour deposits were resistant to DOX whereas subcutaneous (s.c.) tumours were sensitive. The authors determined that the difference in response was not due to differences in DOX distribution or potency but resulted from over expression of mdr1 mRNA in the resistant sites, they made the valid point that human colon cancer xenografts growing subcutaneously in nude mice often respond to DOX, whereas human colon cancer does not. These observations reiterate the requirement for continual target characterization in in vivo models. Since modern molecular biology allows for the dissection of signalling pathways instrumental in cell proliferation and death, the number of potential molecular targets for drug development is increasing. Preclinical systems must be selected or designed in such a way to ensure that the appropriate target is expressed, and the appropriate controls in which the target is down-regulated or missing should be used in order to establish proof of principle and to ensure the validity of the therapeutic mechanism.

### HOST EFFECTS

Although a molecular target approach using high-throughput, robotic cell-free in vitro screens or cell line panels followed by properly characterized in vivo models should increase the efficiency of drug-discovery programmes, novel therapeutic strategies for cancer are clearly not going to be identified only by in vitro screening. These screens do not take into consideration indirect mechanisms such as host metabolism to an active species or immunomodulation. Within the rapidly expanding field of tumour biology several new therapeutic avenues are being explored that address host/tumour relationships or exploit specific features of solid tumours, thus relying even more on clinically relevant preclinical in vivo models.

### TUMOUR BLOOD SUPPLY

One area of solid tumour biology that has been receiving a great deal of attention over recent years is the tumour blood supply. Broadly speaking, to date there are four different strategies attempting to exploit the tumour blood supply for therapeutic gain and all rely not only on differences in the architecture and cellular, biochemical and molecular properties between normal and tumour vasculature but also on the use of a properly characterized model. The major thrust at present seems to be concerned with anti-angiogenesis, i.e. a strategy to prevent the development of new blood vessels and to restrict solid tumour growth. There has been an enormous leap in our understanding of the processes and molecular control of tumour angiogenesis in recent years and the anti-angiogenic approach has been the subject of some excellent comment articles and reviews (Folkman, 1990, 1997; Baillie et al, 1995; Pluda, 1997). Modulation of tumour growth by the use of monoclonal antibodies to vascular endothelial growth factor (VEGF) has proved successful in preclinical studies (Warren et al, 1995) but the rational design of drugs which will specifically interfere with steps in the angiogenic process in tumours is in its infancy as is the development of gene therapy approaches (Kong and Crystal, 1998). However, targets are being identified, so continual development, improvement and characterization of in vivo tumour models in which to test specific strategies is required.

The other attempts to exploit tumour blood supply have concentrated on the existing blood vessels within solid tumours. One approach relies on the lack of smooth muscle and innervation of the new blood vessels growing in solid tumours. Several experimental studies have demonstrated potentiation of the activity of both standard and investigational drugs by combination with a

© Cancer Research Campaign 1999

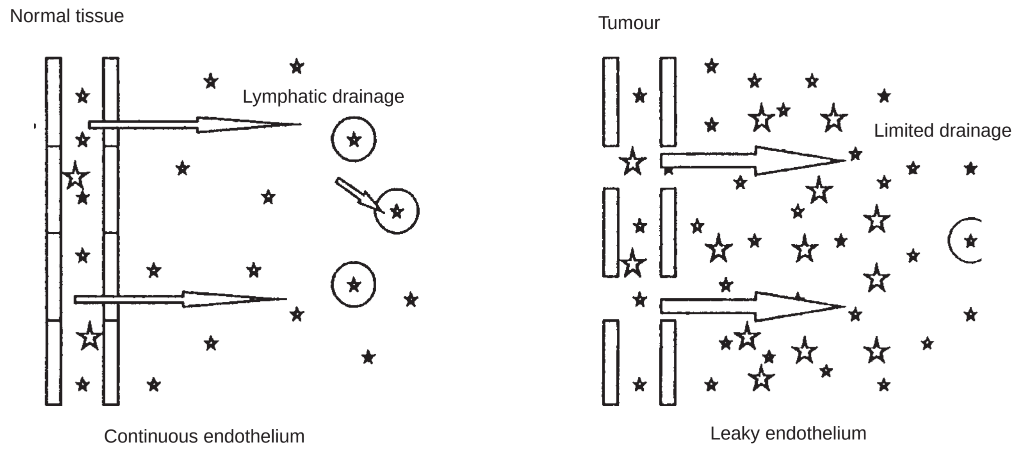

**Figure 1** Diagramatic representation of *E*nhanced *P*ermeability and *R*etention in tumours (Matsumura and Maeda, 1986)

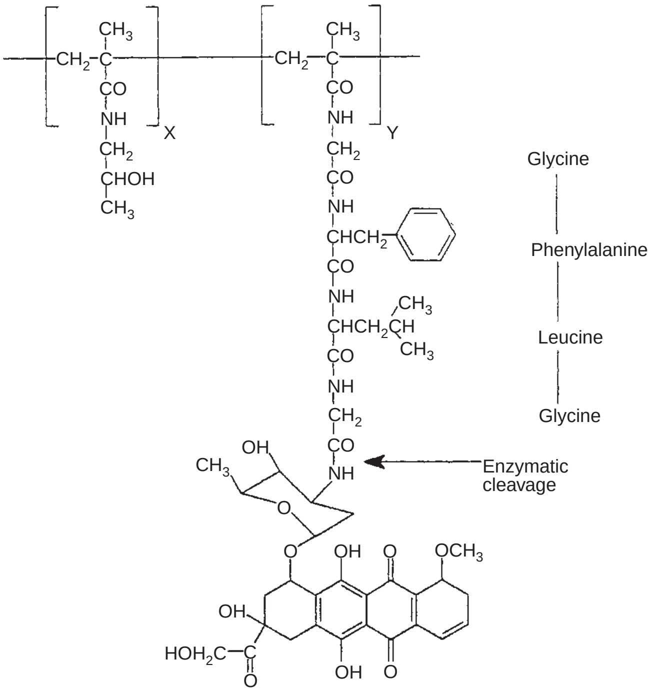

**Figure 2** Chemical structure of PK1 (Duncan, 1992)

variety of vasoactive agents. A large number of agents alter blood flow in tumour models (Hirst and Wood, 1989) but many experimental studies utilized the anti-hypertensive hydralazine which has been shown to decrease blood flow through transplantable tumours in rodents (Chan et al, 1984; Jirtle, 1988). Hydralazine enhanced the effectiveness of bioreductive drugs, such as the nitroimidazole RSU 1069 (Chaplin and Acker, 1987), tirapazamine (Brown, 1987), EO9 (Bibby et al, 1993) and mitomycin C (Cowen et al, 1994) but potentiation was also seen with melphalan (Stratford et al, 1988) and tauromustine (Quinn et al, 1992). Although these and other similar studies indicated a potential therapeutic strategy they have not yet resulted in a clinical advantage. Rowell et al (1990) showed that single-dose oral hydralazine caused the blood flow through human lung tumours to decrease rather than increase. A study by Field et al (1991) demonstrated a difference in the effects of hydralazine on vascular perfusion between a series of primary and subcutaneously transplanted malignancies. The transplanted tumours responded to hydralazine

*© Cancer Research Campaign 1999*

whereas the primary tumour from which they were derived did not. Only 4/11 primary tumours responded to hydralazine. All the tumours used in the preceding papers were transplanted subcutaneously in mice so, although they may have established proof of a particular hypothesis, the studies are unlikely to be clinically predictive. An investigation from this laboratory (Cowen et al, 1995) demonstrated that hydralazine was more effective at shutting down blood supply to s.c. murine colon tumours than to the same tumours transplanted orthotopically, and the same dose of hydralazine was completely ineffective in liver metastasis in this model. These studies highlight the lack of prediction by s.c. tumours for this strategy and the importance of selecting clinically relevant tumour models in appropriate anatomical sites.

Another approach to targeting solid tumours by means of their poorly formed blood vessels relies on the enhanced permeability and retention effect (EPR) coined by Matsumura and Maeda (1986) (Figure 1). Many experimental tumours appear to selectively concentrate macromolecules of around 20 kDa molecular mass. This phenomenon is not due to just the leakiness of the tumour vasculature but also lack of the organized lymphatic drainage that usually occurs in normal tissues. One strategy that seems to be clinically useful at present is the incorporation of cytotoxic moieties into copolymers of hydroxypropylmethylacrylamide (HPMA) (Duncan, 1992). One of these compounds, PK1, is a copolymer with peptidyl side-chains terminating in DOX (Figure 2). The stable side-chain is thought to ensure that there is no extracellular release of DOX so that normal tissue toxicity is reduced. The polymer–drug conjugate was designed so that the intact macromolecule is taken up into cells by pinocytosis and transferred to the lysosomal compartment where it is exposed to an array of enzymes resulting in release of DOX. PK1 containing at least four times the normal clinical dose of DOX was delivered to patients in a phase I trial and two responses were seen (Connors and Pinedo, 1997). This kind of targeting seems a worthwhile strategy to pursue, but it is imperative that appropriate in vivo tumour models are employed in order to ensure the correct molecules are selected rapidly for clinical study. Preclinical studies with PK1 indicated that, not only was it active against vascularized solid tumours in mice, it was also active against L1210 leukaemia when delivered by the intraperitoneal route (Duncan et al, 1992). Recent studies in this laboratory have also demonstrated that tumours in which the blood vessels are not leaky are no more responsive to PK1 than to DOX. This study also demonstrated activity against avascular murine colon cancer lung colonies (Loadman et al, 1998) suggesting that, although there is impelling evidence from numerous studies that EPR plays a major role in the activity of PK1, the absence of such a phenomenon from micrometastases would not preclude activity. Appropriate studies are required to further investigate this interesting approach.

There is evidence emerging that the endothelium of solid tumour vasculature itself may be a useful target for drug therapy. It has been known since the 1930s that colchicine caused anti-vascular effects in experimental tumours (Clearkin, 1937) and it is now clear that some clinically useful tubulin interactive agents possess a vascular component in their mechanism of action against murine solid cancer models (Hill et al, 1993). However, these effects are seen only at doses close to MTD; the therapeutic index is extremely small so the ratio of sensitivity of tumour endothelium to normal cells is also small. A number of stilbenes, the combretastatins, has been isolated from the South African bush willow, *Combretum caffrum*, and based on them a series of synthetic analogues has been synthesized (Pettit et al, 1989, 1995). Combretastatin A4 has been shown to bind to tubulin at the colchicine-binding site (McGown and Fox, 1989) and cause tumour blood flow reduction (Chaplin et al, 1996), and Dark et al (1997) have demonstrated vascular shutdown with combretastatin A4 phosphate prodrug in s.c. experimental tumours at less than one-tenth of the MTD. Fearing the possibility of transplantation artefacts in these anti-vascular effects we have examined the efficacy of both combretastatin A4 and its prodrug on orthotopically transplanted murine colon tumours and ensuing metastatic

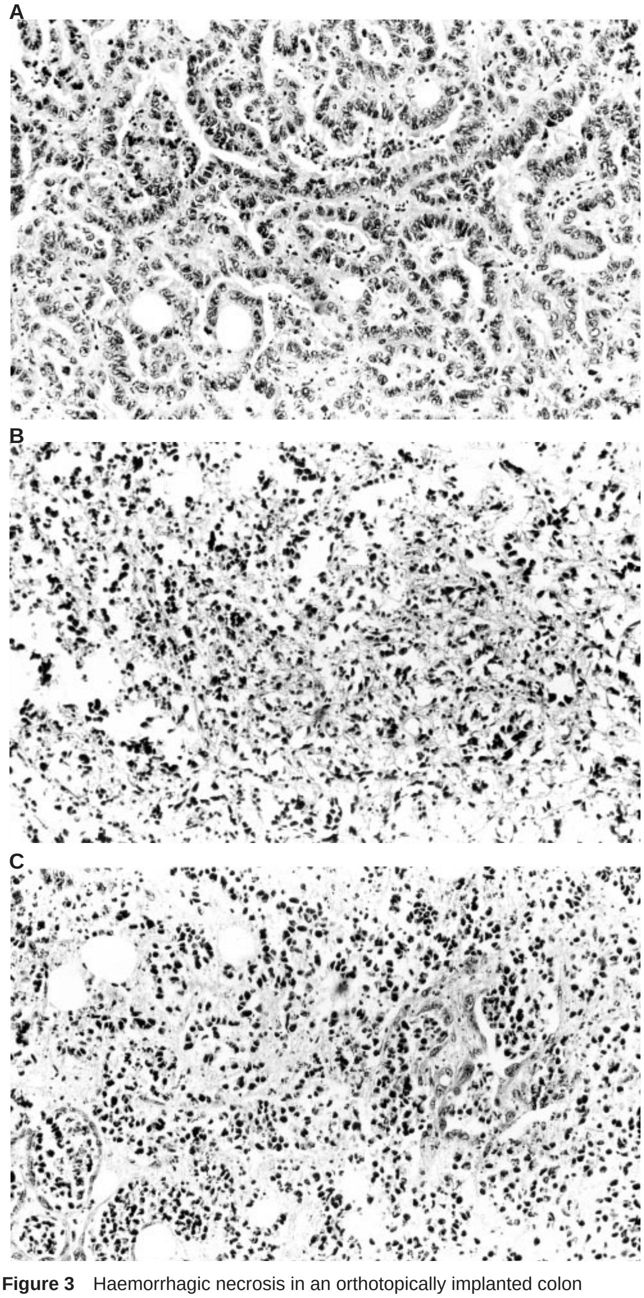

**Figure 3** Haemorrhagic necrosis in an orthotopically implanted colon tumour and its metastasis following treatment with the investigational agent combretastatin A4 (courtesy of K Grosios). (**A**) Control caecal implant; (**B**) treated caecal implant; (**C**) treated metastatic deposit deposits (Grosios et al, 1997). Both parent compound and prodrug caused major haemorrhagic necrosis in tumours at the orthotopic site and in metastatic deposits (Figure 3). Careful examination of morphological effects indicated that lung deposits that had not undergone neovascularization did not respond to either compound, indicating that vascularization and not transplantation site seems to be the important component for the activity of these compounds. The combretastatin A4 prodrug is awaiting phase I clinical trial and it remains to be seen whether the vascular effects predicted from the preclinical models will occur.

Another compound selected for clinical trial on the basis of preclinical anti-vascular effects is 5,6-dimethyl xanthenone acetic acid (XAA). This is a more potent analogue of flavone acetic acid (FAA) (Atassi et al, 1985) from a series developed by Baguley and colleagues in Auckland, New Zealand (Atwell et al, 1989; Rewcastle et al, 1989, 1991*a*, 1991*b*, 1991*c*). FAA is an interesting molecule that had impressive experimental activity but, disappointingly, failed to show any clinical activity (reviewed by Bibby, 1991; Bibby and Double, 1993). XAA again showed impressive haemorrhagic necrosis in vascularized solid tumour models but this time at tenfold lower doses than FAA (Rewcastle et al, 1991*a*; Ching et al, 1992; Laws et al, 1995*a*) and also caused widespread haemorrhagic necrosis in an orthotopic model of human colon cancer (Laws et al, 1995*b*). Phase I clinical trials are ongoing in Auckland and in Mt Vernon Hospital/Gray Laboratory and Bradford, UK. To date, tumour vascular effects have been noted in two patients (Rustin et al, 1998) suggesting that the preclinical models may well have been predictive in this case but, more importantly, demonstrating proof of principle, i.e. that a vascular effect demonstrated in in vivo preclinical models can also occur in solid malignancies in man. These clinical effects suggest that this anti-vascular strategy may well be worth pursuing with other more potent chemical entities.

A particularly interesting development that again relies on thorough characterization of in vivo models has come from the identification that certain peptides home specifically to the vasculature of specific organs (Pasqualini and Ruoslahti, 1996) and to tumour vasculature (Arap et al, 1998). In the latter study the authors have demonstrated that it may be possible to target chemotherapeutic drugs specifically to tumour vasculature on the basis of differential expression of receptors.

### ORTHOTOPIC TUMOUR MODELS

It is now clear from large numbers of studies, some of which are cited here, that it is necessary to take account of not only the relevance of the tumour type utilized for the in vivo evaluation of novel drugs and other therapeutic strategies but also tumour site. In order to do this it is necessary to use model systems that reflect the morphology and growth characteristics of clinical disease but, equally as importantly, must be technically feasible and ethically acceptable to carry out. For the last 20 years good studies have been reported in the literature indicating that such orthotopic systems exist (e.g. Tan et al, 1977; Goldrosen et al, 1986; Bresalier et al, 1987; Morikawa et al, 1988; Fu et al, 1991) and the worth of such systems has been extensively reviewed (Fidler et al, 1990; Fidler, 1991; Gutman and Fidler, 1995; Hoffman, 1997). It is important to remember that, in the case of common solid cancers in humans, the primary tumour mass is usually excised by the surgeon and it is the metastases that require novel therapeutic strategies, so models should reflect these clinical metastases. It is still necessary to refine orthotopic techniques in order to improve the predictability of these models in the future, either by characterization for specific molecular targets within these clinically relevant sites of metastasis, or to develop similar models with tumour cells appropriately transfected to express the target of interest. It must be remembered that, if one adopts an orthotopic approach with models for drug discovery, due attention is given to appropriate end-points. Lethality should not be seen as an alternative to a specific pharmacodynamic end-point such as changes in blood flow in the case of anti-vascular strategies or production of cytokines by immunomodulators etc.

### CONCLUSIONS

It is clear that murine tumour models have been very helpful in determining basic principles of cancer chemotherapy and to date have been instrumental in identifying and evaluating a limited number of clinically useful agents. Contrary to popular belief many murine tumours are not easy to cure with standard chemotherapeutic agents and, if adequate attention is paid to clinically relevant end-points and therapeutic index, they can be good predictors of clinical activity. Of course today a whole host of approaches and assays is available to drug-discovery teams:

- high throughput robotic cell-free screens
- in vitro cell lines (sometimes genetically modified to express a specific target)
- hollow-fibre in vivo assays
- induced rodent tumour systems – usually intraperitoneal or subcutaneous
- spontaneous rodent tumours
- models of metastasis
- orthotopic models
- human tumour xenografts
- transgenic systems.

Modern drug discovery should be much more mechanistically based and target-driven, so the first two of these systems should continue to lead to identification of structures that have potential for development. Assays like the NCI hollow-fibre screen provide a rapid and relatively inexpensive method for assessing in vivo potential. The remaining systems should be carefully selected based on the particular therapeutic approach. The evidence from the literature is clear in that it indicates that rodent tumours should not be used for random screening. It is necessary to fully characterize tumour models to ensure that the precise target for the strategy being evaluated is expressed in vivo. Since targets are known to vary with different anatomical sites, more effort should be placed on developing models in clinically relevant sites rather than the continuous use of subcutaneously transplanted tumours whilst ignoring their tissue of origin or likely site of metastasis in humans. Ultimately, regardless of the molecule being investigated and developed, it is necessary to produce a safe pharmaceutical preparation that can be delivered in an appropriate way to reach the target for which it has been designed, so to make real progress in drug development appropriate in vivo rodent models are still essential. However, in addition to scientific considerations, due ethical consideration must be given to ensure selection of the appropriate model for the task in hand and animal welfare guidelines must be followed throughout.

*© Cancer Research Campaign 1999*

In drug-discovery programmes it is not financially viable, nor does it make scientific sense, to use large panels of murine tumours or human tumour xenografts for extensive in vivo screening, particularly when these xenografts are often poorly characterized. The most efficient and ethical approach must be to design limited, but relevant, experiments to address key questions, e.g:

- Can effective drug concentrations based on previous in vitro data be achieved in vivo?
- Is the molecule reaching its designated clinically relevant target?
- Is there efficacy or, in other words, is there proof of principle?

If these questions can be answered in the affirmative then the compound should be scheduled for clinical trial without the need for further extensive in vivo testing.

In summary, there are certain key requirements for in vivo studies:

- Models must have known clinically-relevant target status.
- Appropriate bioavailability of test molecules should be demonstrated.
- Consideration of therapeutic index is vital.
- The anatomical site of the tumour should be considered.

Proof of principle can be achieved with the use of individually tailored rodent model systems, whether they be syngeneic murine or human tumours, and these should continue to have a key role in selecting those compounds for clinical evaluation that have the best chance of causing a significant therapeutic benefit to the patient. Hopefully the days of screening in large panels of poorly characterized tumours in animals are a thing of the past.

### REFERENCES

Arap W, Pasqualini R and Ruoslahti E (1998) Cancer treatment by targeted drug delivery to tumor vasculature in a mouse model. *Science* **279**: 377–380

Attassi G, Briet P, Berthelon JJ and Colonge F (1985) Synthesis and antitumor-activity of some 8-substituted-4-oxo-4H-1-benzopyrans. *Eur J Med Chem* **20**: 393–402

Atwell GJ, Rewcastle GW, Baguley BC and Denny WA (1989) Synthesis and antitumour activity of topologically related analogues of flavone acetic acid. *Anticancer Drug Design* **4**: 161–169

Baillie CT, Winslet MC and Bradley NJ (1995) Tumour vasculature – a potential therapeutic target. *Br J Cancer* **72**: 257–267

Bibby MC (1991) Flavone acetic acid – an interesting novel therapeutic agent or just another disappointment. *Br J Cancer* **63**: 3–5

Bibby MC and Double JA (1993) Flavone acetic acid – from laboratory to clinic and back. *Anti-Cancer Drugs* **4**: 3–17

Bibby MC, Double JA, Wahed IA, Hirbawi N and Baker TG (1988) The logistics of broader pre-clinical evaluation of potential anti-cancer agents with reference to anti-tumor activity and toxicity of mitozolomide. *Br J Cancer* **58**: 139–143

Bibby MC, Sleigh NR, Loadman PM and Double JA (1993) Potentiation of EO9 anti-tumour activity by hydralazine. *Eur J Cancer* **29A**: 1033–1035

Boyd MR (1989) Status of the NCI preclinical antitumor drug discovery screen. In *Cancer*: *Principles and Practice of Oncology Update*, De Vita VT Jr, Hellman S and Rosenberg SA (eds), vol 3, pp. 1–12. Lippincott: Philadelphia

Bresalier RS, Hujanen ES, Raper SE, Roll FJ, Itzkowitz SH, Martin GR and Kim YS (1987) An animal model for colon cancer metastasis: establishment and characterization of murine cell lines with enhanced liver-metastasizing ability. *Cancer Res* **47**: 1398–1406

Brown JM (1987) Exploitation of bioreductive agents with vaso-active drugs. In *Radiation Research*, of vol. 2, Fieldan et al (eds), pp. 719. Taylor & Francis, London

Chan RC, Babbs CF, Vetter RJ and Lamar CH (1984) Abnormal response of tumor vasculature to vasoactive drugs. *J Natl Cancer Inst* **72**: 145–150

Chaplin DJ and Acker B (1987) The effect of hydralazine on the tumor cytotoxicity of the hypoxic cell cytotoxin RSU-1069: evidence for therapeutic gain. *Int J Radiat Oncol Biol Phys* **13**: 579–585

Chaplin DJ, Pettit GR, Parkins CS and Hill SA (1996) Antivascular approaches to solid tumour therapy: evaluation of tubulin binding agents. *Br J Cancer* **74** (Suppl XXVII): S86–S88

Ching L-M, Joseph WR and Baguley BC (1992). Anti-tumour responses to flavone-8-acetic acid and 5,6-dimethylxanthenone-4-acetic acid in immune deficient mice. *Br J Cancer* **66**: 128–130

Clearkin PA (1937) The effect of colchicine on normal and neoplastic tissues in mice. *J Pathol Bact* **44**: 469–480

Connors TA and Pinedo M (1997) Drug development in Europe. In *Anticancer Drug Development Guide*: *Preclinical Screening, Clinical Trials and Approval*, Teicher B, (ed), pp. 271–288. Humana Press: Totowa, NJ

Collard J, Matthew AM, Double JA and Bibby MC (1995) EO9: relationship between DT-diaphorase levels and response in vitro and in vivo. *Br J Cancer* **71**: 1199–1203

Corbett TH, Valeriote FA and Baker LH (1987) Is the P388 murine tumor no longer adequate as a drug discovery model? *Investigational New Drugs* **5**: 3–20

Cowen SE, Loadman PM, Double JA and Bibby MC (1994) Hydralazine alters murine mitomycin C plasma pharmacokinetics – a possible explanation of drug potentiation. *Br J Cancer* **69** (Suppl XXI): 41

Cowen SE, Bibby MC and Double JA (1995) Characterisation of the vasculature within a murine adenocarcinoma growing in different sites to evaluate the potential of vascular therapies. *Acta Oncol* **43**: 357–360

Dark GG, Hill SA, Prise VE, Tozer GM, Pettit GR and Chaplin DJ (1997) Combretastatin A-4, an agent that displays potent and selective toxicity toward tumor vasculature. *Cancer Res* **57**: 1829–1834

Dong Z, Radinsky R, Fan D, Tsan R, Bucana CD, Wilmanns C and Fidler IJ (1994) Organ-specific modulation of steady-state mdr gene expression and drug resistance in murine colon cancer cells. *J Natl Cancer Inst* **86**: 913–920

Double JA and Bibby MC (1989) Therapeutic Index: a vital component in selection of anticancer agents for clinical trial. *J Natl Cancer Inst* **81**: 988–994

Duncan R (1992) Drug-polymer conjugates: potential for improved chemotherapy. *Anti-Cancer Drugs* **3**: 175–210

Duncan R, Seymour LW, O'Hare KB, Flanagan PA, Wedge S, Hume IC, Ulbrich K, Strohalm J, Subr V, Spreafico F, Grandi M, Ripamonti M, Farao M and Suarato A (1992) Preclinical evaluation of polymer-bound doxorubicin. *J Controlled Rel* **19**: 331–346

Field SB, Needham S, Burney IA, Maxwell RJ, Coggle JE and Griffiths JR (1991) Differences in vascular responses between primary and transplanted tumours. *Br J Cancer* **63**: 723–726

Fidler IJ (1991) Orthotopic implantation of human colon carcinomas into nude mice provides a valuable model for the biology and therapy of metastasis. *Cancer Metastasis Rev* **10**: 229–243

Fidler IJ, Naito S and Pathak S (1990) Orthotopic implantation is essential for the selection, growth and metastasis of human renal cell cancer in nude mice. *Cancer Metastasis Rev* **9**: 149–165

Fidler IJ, Wilmanns C, Staroselsky A, Radinsky R, Dong Z and Fan D (1994) Modulation of tumor cell response to chemotherapy by the organ environment. *Cancer Metastasis Rev* **13**: 209–222

Fitzsimmons SA, Workman P, Grever M, Paull K, Camalier R and Lewis AD (1996) Reductase enzyme expression across the National Cancer Institute tumor cell line panel: correlation with sensitivity to mitomycin C and EO9. *J Natl Cancer Inst* **88**: 259–269

Folkman J (1990) What is the evidence that tumours are angiogenesis dependent. *J Natl Cancer Inst* **82**: 4–6

Folkman J (1997) Antiangiogenic therapy. In *Cancer*: *Principles and Practice of Oncology*, DeVita VT Jr Hellman S, Rosenberg SA (eds), pp. 3075–3085. Lippincott-Raven: Philadelphia

Fu XY, Besterman JM, Monosov A and Hoffman RM (1991) Models of human metastatic colon cancer in nude mice orthotopically constructed by using histologically intact patient specimens. *Proc Natl Acad Sci USA* **88**: 9345–9349

Goldin A and Mantel N (1957) The employment of combinations of drugs in the chemotherapy of neoplasia: a review. *Cancer Res* **17**: 635–647

Goldin A, Venditti JM, Humphreys SR and Mantel N (1956) Modification of treatment schedules in the management of advanced mouse leukemia with aminopterin. *J Natl Cancer Inst* **17**: 203–212

Goldrosen MH, Paolini N Jr and Holyoke ED (1986) Description of a murine model of experimental hepatic metastases. *J Natl Cancer Inst* **77**: 823–828

Grosios K, McGown AT, Pettit GR and Bibby MC (1997) Evaluation of combretastain A4 and its prodrug, in an orthotopic tumour model. *Proc Am Assoc Cancer Res* **38**: 307

*© Cancer Research Campaign 1999*

Gutman M and Fidler IJ (1995) Biology of human colon cancer metastasis. *World J Surg* **19**: 226–234

Hill SA, Lonergan SJ, Denekamp J and Chaplin DJ (1993). Vinca alkaloids: anti-vascular effects in a murine tumour. *Eur J Cancer* **29A**: 1320–1324

Hirst DG and Wood PJ (1989) The control of tumour blood flow for therapeutic benefit. *BIR Rep* **19**: 76

Hoffman RM (1997) Fertile seed and rich soil. In *Anticancer Drug Development Guide*: *Preclinical Screening, Clinical Trials and Approval*, Teicher B (ed), pp. 127–144. Humana Press: Totowa, NJ

Jirtle RL (1988) Chemical modifications of tumour blood flow. *Int J Hyperthermia* **4**: 355–371

Kong H-L and Crystal RG (1998) Gene therapy strategies for tumor antiangiogenesis. *J Natl Cancer Inst* **90**: 273–286

Laws AL, Matthew AM, Double JA and Bibby MC (1995*a*) Preclinical in vitro and in vivo activity of 5,6-dimethylxanthenone-4-acetic acid. *Br J Cancer* **71**: 1204–1209

Laws AL, Matthew AM, Bibby MC and Double JA (1995*b*) The activity of 5,6-MeXAA on a subcutaneous and orthotopic model of human colon cancer. *Br J Cancer* **71** (Suppl XXIV): 40

Loadman PM, Bibby MC, Double JA, Al-Shakhaa WM and Duncan R (1998) PK1 and doxorubicin pharmacokinetics in two colon tumour models with differing responses to PK1. *Proc Am Assoc Cancer Res* **39**: 425

Martin DS (1981) The scientific basis for adjuvant chemotherapy. *Cancer Treat Rep* **8**: 169–189

Martin DS, Stolfi RL and Sawyer RC (1984) Commentary on 'Clinical predictivity of transplantable tumor systems in the selection of new drugs for solid tumors: rationale for a three-stage strategy'. *Cancer Treat Rep* **68**: 1317–1318

Matsumura Y and Maeda H (1986) A new concept for macro-molecular therapeutics in cancer chemotherapy; mechanism of tumoritropic accumulation of proteins and the antitumour agent SMANCS. *Cancer Res* **6**: 6387–6392

McGown AT and Fox BW (1989) Structural and biochemical comparison of the anti-mitotic agents colchicine, combretastatin A4 and amphethinile. *Anticancer Drug Des* **3**: 249–254

Morikawa K, Walker SM, Nakajima M, Pathak S, Jessup JM and Fidler IJ (1988) Influence of organ environment on the growth, selection, and metastasis of human colon carcinoma cells in nude mice. *Cancer Res* **48**: 6863–6871

Muggia FM (1987) Closing the loop: providing feedback on drug development. *Cancer Treat Rep* **71**: 1–2

Newlands ES, Stevens MF, Wedge SR, Wheelhouse RT and Brock C (1997) Temozolomide: a review of its discovery, chemical properties, pre-clinical development and clinical trials. *Cancer Treat Rev* **23**: 35–61

Pasqualini R and Ruoslahti E (1996) Organ targeting in vivo using phase display peptide libraries. *Nature* **380**: 364–366

Pettit GR, Singh SB, Hamel E, Lin CM, Alberts DS and Garcia-Kendall D (1989) Isolation and structure of the strong cell growth and tubulin inhibitor combretastatin A4. *Experientia* **45**: 209–211

Pettit GR, Temple CJR, Nnarayanan VL, Varma R, Simpson MJ, Boyd MR, Rener GA and Bansal NN (1995) Antineoplastic agent 322. Synthesis of combretastatin A4 prodrugs. *Anticancer Drug Des* **10**: 299–309

Phillips RM (1996) Human-DT-diaphorase as a candidate for enzyme-directed bioreductive drug development. *Drugs of the Future* **21**: 1247–1256

Phillips RM, Cronin BP, Jarrett CM and Bibby MC (1998) Distribution of DT-diaphorase in malignant and normal human lung tissue. *Br J Cancer* **78** (Suppl 1): 59

Plowman J, Dykes DJ, Hollingshead M, Simpson-Herren and Alley MC (1997) Human tumor xenograft models in NCI drug development. In *Anticancer Drug Development Guide*: *Preclinical Screening, Clinical Trials and Approval*, Teicher B (ed), pp. 101–125. Humana Press: Totowa, NJ

Pluda JM (1997) Tumor-associated angiogenesis: mechanisms, clinical implications and therapeutic strategies. *Semin Oncol* **24**: 203–218

Quinn PKM, Bibby MC, Cox JA and Crawford SM (1992) The influence of hydralazine on the vasculature, blood perfusion and chemosensitivity of MAC tumours. *Br J Cancer* **66**: 323–330

Rewcastle GW, Atwell GJ, Baguley BC, Calveley SB and Denny WA (1989) Potential anti-tumour agents. 58. Synthesis and structure–activity relationships of substituted xanthenone-4-acetic acid against the colon 38 tumour in vivo. *J Med Chem* **32**: 793–799

Rewcastle GW, Atwell GJ, Zhuang L, Baguley BC and Denny WA (1991*a*) Potential anti-tumour agents. 61. Structure–activity relationships for in vivo colon 38 activity among di-substituted 9-oxo-9*H*-xanthene-4-acetic acids. *J Med Chem* **34**: 217–222

Rewcastle GW, Atwell GJ, Palmer BD, Boyd PDW, Baguley BC and Denny WA (1991*b*) Potential antitumour agents. 62. Structure–activity relationships for tricyclic compounds related to the colon tumour active drug 9-oxo-9*H*-xanthene-4-acetic acid. *J Med Chem* **34**: 491–496

Rewcastle GW, Atwell GJ, Baguley BC, Boyd M, Thomsen LL, Zhuang L and Denny WA (1991*c*) Potential antitumour agents. 63. Structure–activity relationships for side-chain analogues of the colon 38 active agent 9-oxo-9*H*-xanthene-4-acetic acid. *J Med Chem* **34**: 2864–2870

Robertson N, Stratford IJ, Houlcrook S, Carmichael J and Adams GE (1992) The sensitivity of human tumour cells to quinone bioreductive drugs: what role for DT-diaphorase? *Biochem Pharmacol* **44**: 409–412

Rowell NP, Flower MA, McCready VR, Cronin B and Horwich A (1990) The effects of single dose oral hydralazine on blood flow through human lung tumours. *Radiother Oncol* **18**: 283

Rustin G, Galbraith S, Taylor N, Stratford M, Jameson M, Thompson P and Bradley C (1998) Impact on tumour perfusion in the CRC Phase I/II Committee Phase I trial of 5,6-dimethylxanthenone-4-acetic acid (DMXAA). Proc 10th EORTC/NCI Symposium on New Drugs in Cancer Therapy: 126

Segura-Aguilar J, Cremades A, Llombart-Bosch A, Monsalve E, Ernster L and Romero FJ (1994) Activity and immunohistochemistry of DT-diaphorase in hamster and human kidney tumours. *Carcinogenesis* **15**: 1631–1636

Skipper HE, Schabel FM Jr, Bell M, Thomson JR and Johnson S (1957) On the curability of experimental neoplasms. I. A-Methopterin and mouse leukemias. *Cancer Res* **17**: 717–726

Skipper HE, Schabel FM Jr and Wilcox WS (1964) Experimental evaluation of potential anticancer agents. XII. On the criteria and kinetics associated with 'curability' of experimental leukemia. *Cancer Chemother Rep* **35**: 1–11

Smitskamp-Wilms E, Peters GF, Pinedo HM, Van Ark-Otte J and Giaccone G (1994) Chemosensitivity to the indoloquinone EO9 is correlated with DT-diaphorase activity and its gene expression. *Biochem Pharmacol* **47**: 1325–1332

Stevens MFG, Hickman JA, Stone R, Gibson NW, Baig CU, Lunt E and Newton CG (1984) Antitumour imidazotetrazines 1, Synthesis and chemistry of 8-carbamoyl-3-(2-chloroethyl)imidazo [5,1-d]-1,2,3, 5-tetrazin 4(3H)-one, a novel broad-spectrum, antitumour agent. *J Med Chem* **27**: 196–201

Stolfi RL, Stolfi LM, Sawyer RC and Martin DS (1988) Chemotherapeutic evaluation using clinical criteria in spontaneous, autochthonous murine breast tumors. *J Natl Cancer Inst* **80**: 52–55

Stratford IJ, Adams GE, Godden J, Nolan J, Howells N and Timpson N (1988) Potentiation of the anti-tumour effect of melphalan by the vasoactive agent, hydralazine. *Br J Cancer* **58**: 122–127

Tan MH, Holyoke ED and Goldrosen MH (1977) Murine colon adenocarcinoma: syngeneic orthotopic transplantation and subsequent hepatic metastases. *J Natl Cancer Inst* **59**: 1537–1544

Warren RS, Yuan H, Matli MR, Gillett NA and Ferrara N (1995). Regulation by vascular endothelial growth factor of human colon cancer tumorigenesis in a mouse model of experimental liver metastasis. *J Clin Invest* **95**: 1789–1797

Workman P, Twentyman P, Balkwill F, Balmain A, Chaplin D, Double J, Embleton J, Newell D, Raymond R, Stables J, Stephens T and Wallace J (1998) United Kingdom Co-ordinating Committee on Cancer Research (UKCCCR) Guidelines for the Welfare of Animals in Experimental Neoplasia, (2nd Edn). *Br J Cancer* **77**: 1–10

*© Cancer Research Campaign 1999*
